# 11.D. Workshop: Depression comorbidity in prediabetes and diabetes: evaluating risk factors and outcomes

**DOI:** 10.1093/eurpub/ckac129.699

**Published:** 2022-10-25

**Authors:** Organised by: Tuebingen University (Germany), Chair persons: Norbert Schmitz (Germany)

## Abstract

Depression is a common comorbidity in prediabetes and diabetes. Evidence for the interaction of depression and behavioural and biological risk factors for (pre-) diabetes and diabetes-related outcomes will be presented and discussed using longitudinal data from the Lifelines Cohort, the Canadian Longitudinal Study on Aging, and the Cartagene study. In addition, digital phenotyping will be discussed as an alternative approach for the assessment of mobility and behaviour in people with diabetes.

**Key messages:**

• Behavioural, psychosocial and biological risk factors might increase the risk of poor diabetes outcomes in a synergistic way.

• Prevention program for diabetes and diabetes realted outcomes should focus on behavioural, psychosocial and biological risk factors simultaneously.

